# The Uræmic Convulsions of Pregnancy, Parturition, and Childbed

**Published:** 1859-02

**Authors:** 


ART. VIII.—*The Uræmic Convulsions of Pregnancy, Parturition, and Childbed.* By Dr. CARL R. BRAUN, Professor of Midwifery, Vienna. Translated from the German, with notes, by J. MATHEWS DUNCAN, F. R. C. P. E., Lecturer on Midwifery, etc., etc. New York: SAMUEL S. & WILLIAM WOOD, 389 Broadway. 1858.

The little book which we have before us is a valuable chapter of a valuable work, and is likely to do much good, in giving to the profession the correct principles of the causation, diagnosis, and treatment of puerperal convulsions. It is a translation of a single chapter of Dr. Braun's work on obstetrics, which has lately been published at Vienna. The high position of the author among obstetrical writers, as well as the patient and well rewarded investigations which he has made in the disease under consideration, and its connection with "uræmic intoxication," render it a most valuable addition to our list of translations, and lay the profession under obligations to the translator. Fortunately, it is seldom the fate of the general practitioner to meet with this fearful disorder; but when he does, his mind is harrassed, if it be not entirely made up in regard to the treatment of this malady, by the opposite measures, which are recommended by the highest authorities, and practiced by intelligent physicians. Some say, bleed, bleed, and bleed again, till the convulsions cease. Others, equally authoritative, deny the benefits of extensive depletion, and rely upon the inhalation of chloroform. Both methods have active supporters; and both, we are compelled to say, in a measure, are sustained by experience.

We regret that such a number of works are on our table, waiting for review, that we will be unable to go into a discussion of the author's views on this important subject, but will be compelled to give them merely the slightest passing notice, and refer our readers to the book itself. Here, the subject is nearly exhausted, and no where will the obstetrical student find it handled in an abler manner, and by a higher authority in this disease.

The author commences with an account of the symptoms of uræmic eclampsia. This need not detain us; suffice it to say, that the disease is faithfully and graphically described. When we come, however, to the premonitory symptoms and the causation, we find the matter fresher and more interesting. The author takes the decided ground, that eclampsia, or puerperal convulsions, is produced by a disease of the kidney, characterized by the presence of albumen in the urine, a congestion of the substance of the gland, which is attended by the exudation of the albuminous and fibrinous elements of the blood, and a want of power in eliminating the urea. This is induced by a venous congestion of the kidney, resulting from the pressure of the gravid uterus on the gland and its vascular appendages. The convulsions, however, do not depend upon the presence of urea in the blood, but upon the carbonate of ammonia, which is formed from the urea, through the influence of a ferment. These points we do not propose to discuss. It is sufficiently well established, however, that albumen is found in the urine of those who are attacked with puerperal convulsions; some authors have asserted that this resulted from the convulsions, but the view entertained by our author, that uræmia is the cause of the convulsions, is undoubtedly the correct one, and albuminuria, indeed, in many cases, preceeds the development of the convulsions by weeks. We also have the symptoms of uræmic intoxication, such as headache, buzzing in the ears, imperfect vision, etc., before the development of eclampsia.

We pass over the pathological anatomy, differential diagnois, etc., and come to the prognosis. Uræmic eclampsia, is, as every one knows, an exceedingly formidable disease. According to Dr. Braun, 30 per cent of the cases have proved fatal. If the patient recover, the albumen disappears from the urine in a few days. An accident which may occur, is hemiplegia, which of course indicates an extraversation of blood in the brain. As would be naturally supposed, the fœtus is in danger so long as it is exposed to the poisoned blood of the mother, and very often it is born dead.

We now come to the section in which we take the deepest interest, namely, that which considers the treatment. Dr. Meigs of Philadelphia, teaches his class to bleed, and that with the boldest hand; sixty ounces; one hundred ounces; if that does not arrest the paroxysm, let it be repeated again and again. Dr. Braun, however, will not admit of such treatment; he considers that bleeding not only is not productive of good, but that it does great harm. This is now quite a common opinion; but we are rather disposed to agree with the translator, who says that his respect for some authorities will not permit him to join in the author's unqualified condemnation of the venesection, though he by no means recommends it. It is certain that we have had, and have now the testimony of careful and intelligent observers in its favor. Chloroform is the great remedy recommended by the author: this, in connection with cathartics, and cold to the head, constitute the important points of medical treatment. He regards it as a very important point, to hasten the expulsion of the contents of the womb; and in this, we believe, he is sustained by all authorities.

We are compelled, in keeping ourselves within the limits which we proposed for this notice, to pass over the other points of treatment, both relating to the disease itself and to its premonitory symptoms, without discussion or criticism. We have seen nothing in the work, with the exception, perhaps, of the theory of the almost universal production of uræmia by the pressure of the gravid uterus upon the renal veins, and the denunciation of blood-letting, which is not now approved by most obstetricians. It is a useful book, and should be in the library of every practitioner.

The work has been issued in a neat duodecimo of 182 pages by the Messrs. Wood of New York, and will be sent by them to those who may desire to purchase it, upon the receipt of $1.

